# Nitrogen deposition homogenizes moss-microbiomes and associated nitrogen fixation but with host-specific responses

**DOI:** 10.1128/aem.00828-26

**Published:** 2026-06-15

**Authors:** Amanda Maria Rydgren Thomsen, Danillo Oliveira Alvarenga, Kathrin Rousk

**Affiliations:** 1 Department of Biology, Center for Volatile Interactions, University of Copenhagen 4321, Copenhagen, Denmark; The University of Tennessee Knoxville, Knoxville, Tennessee, USA

**Keywords:** nitrogen fixation, microbiomes, nitrogen deposition, forest ecosystems, acetylene reduction, mosses, cyanobacteria

## Abstract

Moss microbiomes contribute significantly to terrestrial nitrogen (N) cycling through N fixation, especially in nutrient-poor environments. Yet, the relative roles of environment (abiotic factors) and host species (biotic factors) in shaping these microbiomes remain understudied even though this could reveal how host-specific moss-associated microbiomes are. To assess whether moss-associated microbiomes are shaped predominantly by the moss host or by the environment, we compared three common moss species (*Hylocomium splendens*, *Pleurozium schreberi*, and *Sphagnum* sp.) from ecosystems that differ in atmospheric N deposition rates: a temperate forest with >15 kg N ha^−1^ yr^−1^ and a boreal forest with 2 kg N ha^−1^ yr^−1^ of deposition. We further tested if moss-associated microbiomes and N fixation rates become more similar between the boreal and temperate mosses upon N additions to the boreal mosses. Nitrogen fixation, measured as acetylene reduction, was overall higher in the boreal mosses than in the temperate mosses. Microbial community composition, assessed with 16S rRNA gene sequencing, showed that both environment and host species influence the moss microbiome, but that the environment exerts the stronger influence. Nitrogen additions significantly reduced N fixation rates and changed the composition of moss microbiomes substantially, causing the microbiomes of two of the three investigated mosses to become more similar to each other. Our findings show that the environment (i.e., forest type with different N deposition rates) exerts a strong influence on the moss microbiome and that N deposition favors certain moss-associated microorganisms but lowers N input via moss-associated N fixation considerably.

**IMPORTANCE:**

Mosses host diverse microbiomes that contribute to terrestrial N cycling through processes such as nitrogen (N) fixation. Thus, these microbial communities play important roles in nutrient-poor environments where mosses often dominate. Here, N fixation is a key source of N input, making it important to understand what drives microbiome composition and associated N fixation activity. We ascertained the roles of the environment and moss-host species in shaping the moss-microbiomes and associated N fixation rates in two forest types with varying N deposition rates. We showed that forest type shapes moss-associated microbiomes and N fixation activity, while aligning N availability also aligned N fixation activity and changed the moss microbiomes with implications for the ecosystem functions they provide.

## INTRODUCTION

Nitrogen (N) is an essential nutrient that often limits net primary productivity in nutrient-poor ecosystems such as boreal forests. Even though N_2_ makes up about 78% of the atmosphere, it is largely inaccessible in this form. One way in which it can enter ecosystems is through biological N fixation (BNF), which is the conversion of N_2_ to biologically available ammonia (NH_3_). This is carried out by N-fixing microorganisms (diazotrophs), via the nitrogenase enzyme, which occurs in different isoforms with iron, molybdenum, or vanadium as co-factor (1). Diazotrophs can be free-living in soil, litter, or deadwood, or they can associate with plants, such as mosses (2). In boreal forests, mosses are especially abundant and cover large proportions of the forest floor. Here, primary productivity is often limited by N, with N deposition rates below 2 kg N ha^−1^ yr^−1^, and where moss-associated N fixation is one of the key N input sources, accounting for up to 50% of total ecosystem N input (3, 4). However, moss-associated N fixation is inhibited by high levels of N (5) with a magnitude of inhibition that depends on factors such as moss species, addition rate, and background N levels (6). Previous work showed that only 4 kg of inorganic N ha^−1^ yr^−1^ inhibited N fixation in the ubiquitous moss *Pleurozium schreberi* from boreal forests but not in the bog-forming moss *Sphagnum capillifolium* (6), whereas additions of 30 kg of N ha^−1^ yr^−1^ inhibited N fixation rates uniformly across all investigated moss species from a tropical forest within a day (7). This observed inhibition is not only an end-product inhibition of the enzyme, but the diazotrophs (e.g., cyanobacteria) also disappear from mosses treated with N (8). However, it is unclear how increased levels of N (both via anthropogenic activity and due to natural differences in N availability) impact other members of the moss-microbiome beyond diazotrophs. While the diversity of soil bacterial communities was shown to become lower with increased N levels (9), such assessments are lacking for microorganisms colonizing mosses.

In addition to nutrient availability, host species can also play a role in driving N fixation rates (e.g., 10). For instance, moss host identity was shown to be a better predictor of N fixation rates than environmental factors such as mean annual precipitation, pH, temperature, and tree density, resulting in N fixation rates being twice as high in the moss species *Hylocomium splendens* than in *P. schreberi* even though they grow in the same habitat (11, 12). On the other hand, the morphologically and phylogenetically more distant moss *Sphagnum* sp. has even higher N fixation rates than, for example, *H. splendens* (13). Host species differences in associated N fixation rates have been linked to different morphologies of the moss host; in particular, traits related to hydrology have been put forward as key drivers (12). Besides N fixation, moss host species also plays a role in shaping the associated microbiome, which, in turn, can affect N fixation rates (e.g., 14). For example, an earlier study found that host species identity, and not environmental conditions, was the best predictor of microbial community composition in 26 different moss species from Alaska (15). In contrast, another study showed that moss-associated microbial communities shifted after transplanting the same moss species across two different boreal forests, suggesting that environment plays a larger role than host species (16).

Ecological assembly of microorganisms, including those colonizing mosses, can be driven by deterministic (e.g., species traits, interspecies interactions, environmental filtering) or stochastic (e.g., dispersal limitation, drift) mechanisms (17). The relative importance of these mechanisms may differ between habitats, which could explain the contrasting findings of the specificity of the moss microbiomes (e.g., dispersal limitation depends on the geographic scale investigated). As deterministic factors, both the environment (representing the potential niche) and the moss host (representing the realized niche) could lead to habitat- and moss-specific microbiomes (thus homogenizing microbiomes), while drift as a stochastic mechanism could lead to random changes in community structure and composition (18, 19). Nitrogen fixation, on the other hand, is more plastic since it can be switched on and off depending on N availability within hours (6, 7). However, different diazotrophs associated with different moss species have different N fixation capacities (5), which directly links function to structure.

Few studies have investigated the links between moss microbiomes and N fixation activity across ecosystems, and most studies focused on a single moss species or a single region. Thus, it remains unclear whether the environment or host species plays the dominant role in shaping the moss microbiome and associated N fixation rates, and whether environmental change (such as N availability) can homogenize moss-associated microbiomes. To address these knowledge gaps on how plastic moss-microbiomes and associated N fixation activity are, we compared microbial communities and N fixation rates associated with the same moss species collected from two forests with different N deposition rates (a temperate forest with high N deposition near Copenhagen, Denmark, vs a boreal forest with low deposition in northern Sweden) and assessed the effects of ammonium nitrate additions on N fixation and the microbiome in the three different moss species (*H. splendens*, *P. schreberi,* and *Sphagnum* sp.). Our overall hypothesis was that we would find larger differences in moss-associated N fixation and microbiomes between forests than between moss species within the same forest based on similar dispersal potential within a forest and strong environmental filtering (N availability). More specifically, we hypothesized that (H1) N fixation will be highest in *Sphagnum* sp. followed by *H. splendens* and lowest in *P. schreberi*, with higher rates in mosses from the boreal forest than from the temperate forest; (H2) the environment (i.e., forest type with different levels of N) will have a stronger influence on the moss microbiome than moss species, and thus, mosses from the same forest (boreal or temperate) will have more similar microbiomes than mosses of the same species across forests; and (H3) after adding N, N fixation rates and microbiomes will become homogenized across samples from different forests (boreal and temperate).

## RESULTS

### Nitrogen fixation rates between forests and species

**Fig 1:**
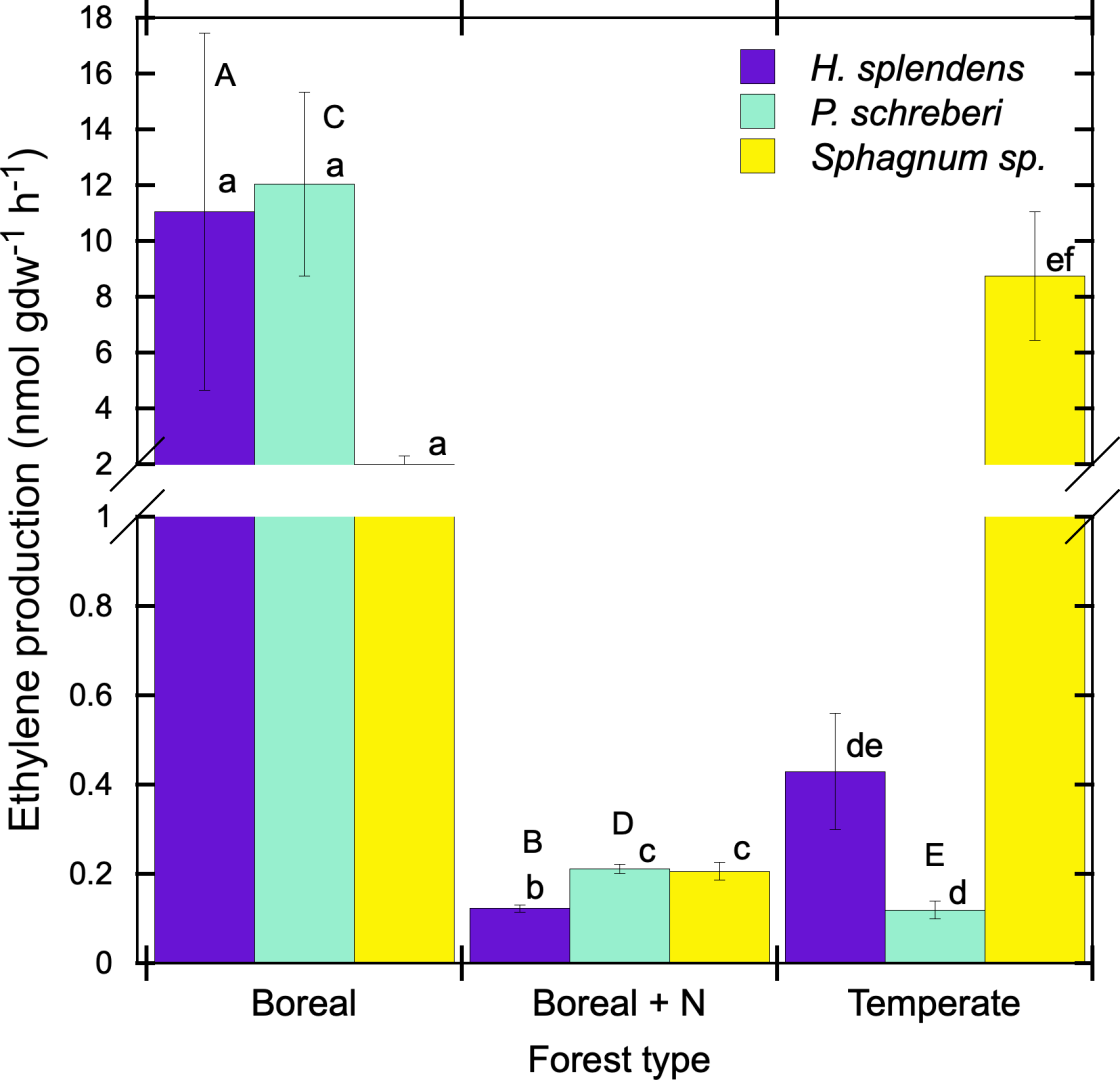
Mean ethylene production rates (nmol ethylene g dw^−1^ h^−1^, ±SE) associated with boreal mosses, boreal mosses treated with ammonium nitrate, and temperate mosses, and of different moss hosts (purple = *H. splendens*, turquoise = *P. schreberi,* and yellow = *Sphagnum* sp.). Lower case letters indicate significant differences (*P* < 0.05) within a forest type, and upper case letteres indicate significant differences within moss species across forest types (*n* = 12 per species for the boreal samples, *n* = 6 for the temperate samples).

We found significant differences in N fixation rates between forest types as well as between moss species. Nitrogen fixation activity (measured as ethylene production) was five times higher in the boreal mosses (mean across all species: 14.79 ± 3.0 nmol g dw^−1^ h^−1^ (SE)) than in the temperate mosses (2.77 ± 1.1 nmol g dw^−1^ h^−1^) (Mann-Whitney *U*, *W* = 107, *P* < 0.01), and ethylene production was significantly higher in *Sphagnum* sp. than in *P. schreberi* in the temperate forest (Kruskal-Wallis, *x*^2^ = 12.21, df = 2, *P* < 0.01) (Fig. 1), confirming H1. No significant differences were found between moss species in the boreal forests (Fig. 1), only a tendency for lower activity in *Sphagnum* sp*.*, while there were significant differences between moss species in the temperate forest. This indicates an interaction between environment and moss host in shaping N fixation activity.

### Bacterial community composition between forests and moss species

**Fig 2:**
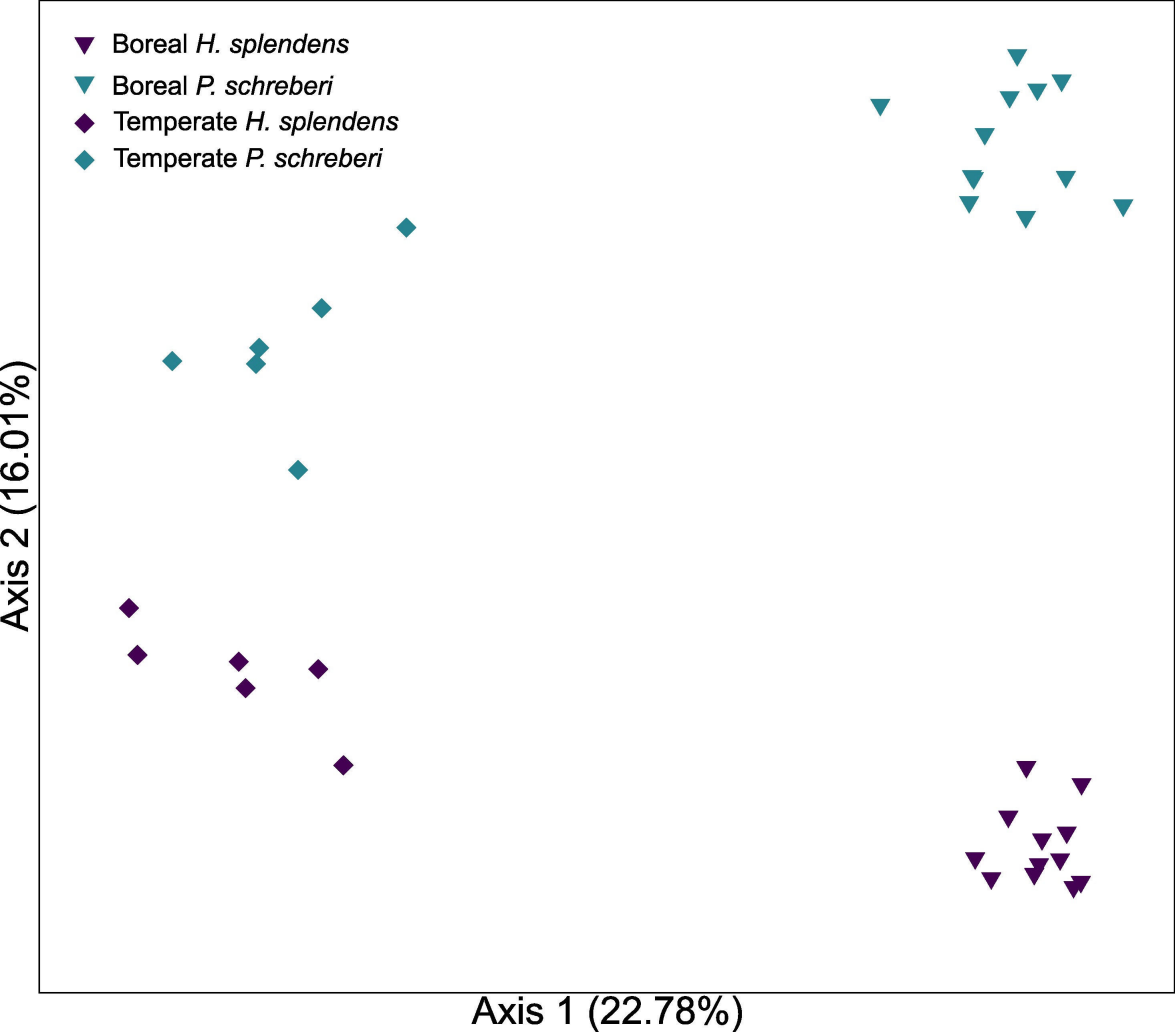
Principal Coordinates Analysis (PCoA) plot based on Bray-Curtis dissimilarity between the bacterial communities associated with boreal and temperate mosses. Each point in the ordination represents the bacterial community of an individual moss sample. Moss species are indicated by color (purple = *H. splendens,* teal = *P. schreberi*), and forest type/environment is indicated by the shape (diamond = boreal, triangle = temperate; *n* = 36).

**Fig 3:**
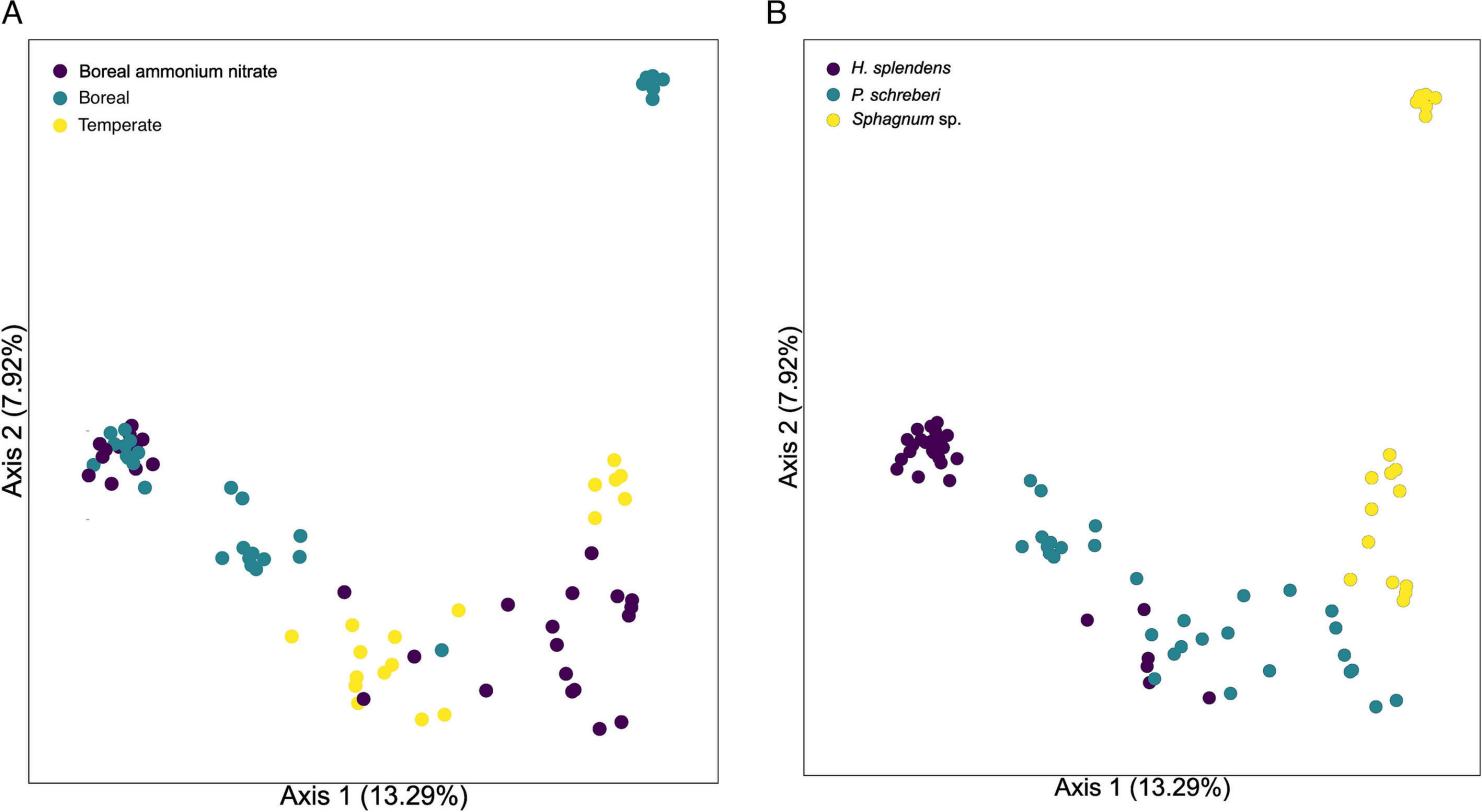
Principal Coordinates Analysis (PCoA) plot based on Bray-Curtis dissimilarity between the bacterial communities associated with boreal and temperate mosses. Each point in the ordination represents the bacterial community of an individual moss sample. (**A**) Differences between forest types and samples that received ammonium nitrate. Color indicates forest and/or addition (purple = boreal ammonium nitrate treated mosses, teal = boreal mosses, and yellow = temperate mosses). (**B**) Differences between moss species (purple = *H. splendens*, teal = *P. schreberi*, and yellow = *Sphagnum* sp.; *n* = 30 for the boreal samples, *n* = 18 for the temperate samples).

We obtained a total of 15,650,892 reverse reads, with 7,014,486 passing quality control. A number of 31,595 ASVs were identified in our samples. Microbial communities associated with the two mosses that were identified to species level (*H. splendens* and *P. schreberi*) were significantly different between moss species as well as between the two forest types (Fig. 2). When *Sphagnum* sp. was excluded from the analysis, forest type drove the differences in microbial communities (pseudo-F = 9.73, *P* < 0.01) and not moss species (*H. splendens* and *P. schreberi*; pseudo-F = 6.01, *P* < 0.01; Fig. 2; Table 1). The Principal Coordinates Analysis (PCoA) plots based on Bray Curtis distance showed differences in community composition by both forest type along axis 1 (22.78%) and moss species along axis 2 (16.01% of the variation explained) (Fig. 2). When including *Sphagnum* sp. in the analysis, species becomes more important in driving differences in microbial community composition between mosses (pseudo-F = 9.85, *P* < 0.01) than between the forest types (pseudo-F = 8.05, *P* < 0.01; Fig. 3A and B; Table 1). The two feather mosses (*H. splendens* and *P. schreberi*) had more similar communities compared to *Sphagnum* sp. (Table 1).

**TABLE 1:** Results from PERMANOVA tests based on Bray Curtis dissimilarity analyses comparing species (*H. splendens*, *P. schreberi*, and *Sphagnum* sp.) and forest types (boreal and temperate) and treatment combinations (boreal, temperate, and boreal treated with ammonium nitrate)^*a*^

Factor	Pairwise	Pseudo-F	*P*
Species^*b*^		9.85	<0.01
	*H. splendens—P. schreberi*	7.14	<0.01
	*H. splendens—Sphagnum* sp.	13.72	<0.01
	*P. schreberi—Sphagnum* sp.	8.23	<0.01
Forest^*b*^	Boreal—Temperate	8.05	<0.01
Species^*c*^	*H. splendens—P. schreberi*	6.01	<0.01
Forest^*c*^	Boreal—Temperate	9.727	<0.01
Addition^*b*^		4.9	<0.01
	Boreal—Temperate	6.24	<0.01
	Boreal—Boreal ammonium nitrate	4.00	<0.01
	Temperate—Boreal ammonium nitrate	4.80	<0.01

^*a*^ We ran the test with and without Sphagnum as this moss was not identified to species level, thus “species” effect cannot be applied to this moss when comparing forest types. A higher pseudo-F suggests greater difference between the groups.

^*b*^ Including *Sphagnum* sp.

^*c*^ Excluding *Sphagnum* sp.

**Fig 4:**
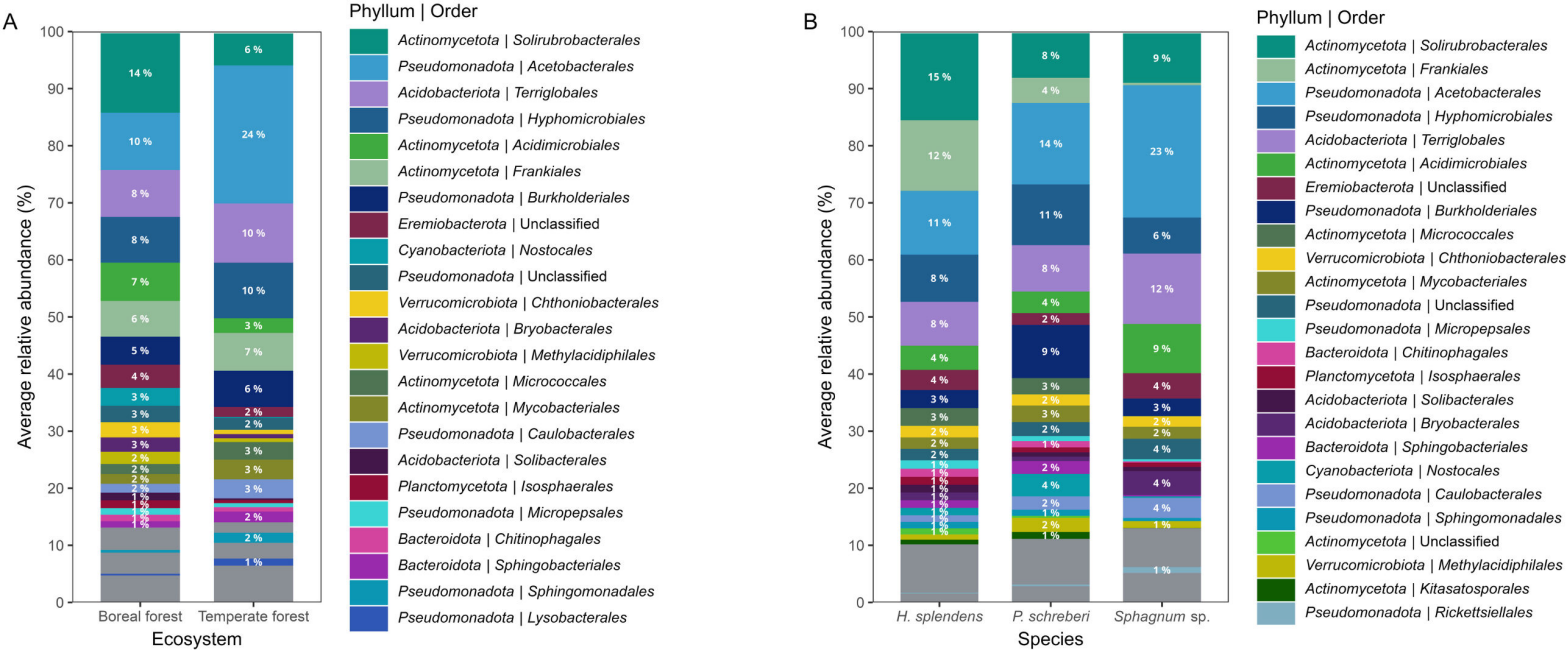
Relative abundances for the most common bacterial orders associated with (untreated) mosses in northern Europe. Bacterial orders are grouped by either forest of origin (**A**) or host species (**B**). Numbers inside the colored bars represent average relative abundance for orders showing percentages of at least 1% in the indicated factor. Orders that presented relative abundances lower than 1% in all factors are colored in gray. Taxonomic identities were estimated based on 16S rRNA gene metabarcoding and sequence comparisons to the SILVA database v. 138. Sequences that did not present significant similarity with previously described orders were reported as “unclassified.”

*Pseudomonadota* (synonym “*Proteobacteria*”) constituted an average of 38.84% of the microorganisms colonizing the mosses and was thereby the most abundant phylum (Fig. 4A and B; Fig. S1a and b). Out of these, especially the order *Hyphomicrobiales* (synonym “*Rhizobiales*”) was common in *Sphagnum* sp. mosses. The second most common phylum was *Actinomycetota*, which constituted 30.09% of the microorganisms on the mosses. The third most common phylum was *Acidobacteriota*, which constituted 10.77% of the microorganisms associated with the mosses, and these were especially abundant in boreal *Sphagnum* sp. mosses, where they constituted up to 21.43%. Only 1.76% average relative abundance were assigned to cyanobacteria across all mosses (Fig. S1a). However, the boreal *P. schreberi* mosses hosted more cyanobacteria (19.00%) compared to the other species and forest (Fig. 4B; Fig. S1b). No significant link between relative abundance of diazotrophs (Nostocales, Hyphomicrobiales, Frankiales) and ethylene production was found, except for a weak negative link with Burkholderiales (*P* = 0.05; rho = −0.15; data not shown). These results somewhat confirm H2 that mosses from the same forest type will have more similar microbiomes, but when including a phylogenetically distant moss (*Sphagnum* sp.), this effect is lessened.

### Moss-associated nitrogen fixation and microbial community composition after nitrogen additions

After adding N, ethylene production as a measure of N fixation dropped significantly in the boreal mosses compared to the non-treated boreal mosses and the temperate mosses (Kruskal-Wallis, *x*^2^ = 10.74, df = 1, *P* < 0.001) (Fig. 1). Ethylene production dropped most in *H. splendens* compared to the other two mosses after N was added (Kruskal-Wallis, *x*^2^ = 12.21, df = 2, *P* < 0.01) (Fig. 1). These results confirm a strong environmental, in this case N availability, control of moss-associated N fixation activity (H3).

Our PCoA plots based on Bray Curtis distance showed clear differences in community composition by forest type and N addition (*P* < 0.01; Table 1). After adding N, they converged slightly (pseudo-F = 4.80, *P* < 0.01) but with moss species-specific effects. Notably, the microbial community associated with the boreal, N treated *Sphagnum* sp. aligned more with the temperate *Sphagnum* (Fig. 3A and B), while the communities associated with *P. schreberi* changed to a completely new composition. In contrast, microbial communities associated with *H. splendens* exposed to N remained similar to the untreated, boreal samples. Thus, even though there are significant differences in community composition after adding N, those differences are less pronounced than those between communities from the boreal and temperate forests (pseudo-F = 4.00, *P* < 0.01; Fig. 3A). Remarkably, cyanobacterial N-fixers disappear from *P. schreberi* after N additions. Moreover, *Bacillota* (synonym “*Firmicutes*”) became more prominent after the ammonium nitrate treatment, especially in *Sphagnum* sp. mosses, where they constituted up to 70.71%, while the average for all the mosses was 2.31% (Fig. S1a). The microbiomes changed and aligned after N additions in two out of the three investigated moss species, indicating an environmental control (H3). However, *H. splendens* microbiomes were not affected by N additions, highlighting a stronger host-control in this moss species.

## DISCUSSION

We set out to assess which factor is more important in driving moss-associated N fixation rates and microbiomes: the moss host or the environment. We focussed on N availability as key driver of N fixation activity. For this, we collected three common moss species from forests with different N status. We could identify only two of the three mosses to species level; therefore, we can only assess the effects of the two known species, *H. splendens* and *P. schreberi*, on associated N fixation rates and microbiomes. Since these two mosses are both feather mosses from the same family (Hylocomiaceae), share similar physiology and morphology, but have commonly different N fixation rates, these two species provide the perfect arena to ascertain moss host vs environmental influence.

### Nitrogen fixation across species and forest types

In accordance with our first hypothesis (H1), overall N fixation rates associated with boreal mosses were higher than in temperate mosses. For instance, N fixation rates in *P. schreberi* and in *H. splendens* were more than five times higher in the boreal forest compared to rates in the same species from the temperate forest. This confirms numerous previous findings that high N levels inhibit N fixation activity (5, 6). This pattern was not found for *Sphagnum* sp., however. The surprisingly low N fixation rates in *Sphagnum* sp. from the boreal forest could simply be due to the fact that the mosses were dry at time of collection and *Sphagnum* is less tolerant to drying-rewetting events than the other two mosses that could have impacted associated diazotroph activity (16, 20).

In contrast, *Sphagnum* sp. had higher N fixation rates than *P. schreberi* collected from the temperate forest, which is in accordance with our hypothesis, as *Sphagnum* mosses are known to support higher N fixation rates than, for example, *P. schreberi* (5, 21). This is likely because *Sphagnum* has higher water holding capacity than other mosses (20, 22), which is a limiting factor for moss-associated N fixation (5). Additionally, diazotrophs associated with *Sphagnum* moss reside not only on the leaf surfaces but also inside water-filled cells that may protect them from variation in environmental factors (23, 24). In the boreal forest, no differences in N fixation rates between moss species were found, which contradicts our first hypothesis but suggests a strong environment effect (forest types with different N levels) on moss-associated N fixation rates. This contrasts with an earlier study that found moss taxonomic family was a better predictor of N fixation in Alaskan mosses than environmental factors such as mean annual precipitation and temperature (11). The contrasting results could be due to similar environments assessed in the Alaskan mosses that do not experience a large enough range of environmental conditions, whereas in our study, mosses were collected from completely different forests in terms of N levels, forest type, and climate. Furthermore, stochastic processes such as colonization and dispersal limitation could be more important in our cross-forest comparison than in the Alaskan study. Nonetheless, N fixation activity was more similar between moss species within a forest than within a species across forests, suggesting a strong environmental control on N fixation rates.

### Influence of the environment and species on the microbiome

Our second hypothesis stated that the environment (i.e., forest type with different N status) influences the microbiome more than moss species (H2). The apparent importance of moss species was driven by the large differences between *Sphagnum* sp. and the other two mosses. When comparing only *H. splendens* and *P. schreberi*, the environment became more important in driving the associated microbiomes than moss identity. Given that *Sphagnum* occupies very different habitats (i.e., waterlogged, acidic environments) and harbors its microbiome endophytically and epiphytically compared to the other two mosses whose microorganisms live epiphytically only, it is not surprising that the associated microbiomes are very different from the two ecologically and phylogenetically more similar feather mosses. When focussing on these two feather mosses only, the associated microbial communities were clearly separated by forest type, which could reflect N availability in the environment (i.e., the communities from the high N forest cluster to the left in Fig. 2), while the second axis could be related to moss traits that differ and lead to different communities (12). This suggests that the environment, and especially factors related to N availability, plays the dominant role in structuring the microbiome in closely related moss species, while host species becomes more important in taxonomically distant mosses (e.g., axis 1 [13.3%] in Fig. 3 could be linked to moss species, whereas axis 2 [7.9%] is likely driven by N). Similar conclusions have been drawn in an earlier study, which found that the environment played a more dominant role than moss species in a boreal forest community (16). Contrastingly, another study found that environmental variables had significant, but subtle effects on the microbiome, while moss species was a stronger predictor of microbial communities (14, 15). This suggests that the relative importance of each factor depends on the degree of environmental contrast and differences in phylogeny and morphology of the host species assessed.

Surprisingly, we did not find a high relative abundance of cyanobacteria in our moss samples, nor did we find any link between the relative abundance of cyanobacteria and N fixation (Fig. 1). This challenges the traditional view of cyanobacteria as principal moss-associated diazotrophs (2) but is in line with other findings that, for example, *Pseudomonadota* can be associated with high N fixation in mosses (25). An earlier study suggested that there are more non-cyanobacterial diazotrophs in association with boreal mosses than previously recognized (15), which may also be the case here, as the most common microbial phylum we found was *Pseudomonadota*. Especially in *Sphagnum* mosses with a very different physiology than the other mosses, only 6% of *nif*H sequences were assigned to cyanobacteria, while 82% were assigned to *Pseudomonadota*, whereof most of these were assigned to *Hyphomicrobiales*. Indeed, *Hyphomicrobiales* was the third most common order of bacteria in our data set. These findings suggest that N fixation is not only driven by cyanobacteria, but rather the net activity of multiple taxa that can be active under different environmental conditions (14). However, our results are based on 16S amplicons and not specific diazotroph markers (i.e., nif*H*). Thus, our links between N fixation and relative abundance of diazotrophs should be taken with caution as our data include potential as well as confirmed diazotrophs.

In a recent study that identified diazotroph communities associated with the same two mosses investigated here (*H. splendens, P. schreberi*) along a steep precipitation gradient in the Arctic, the N-fixer communities differed significantly between the moss species, much more than between location along the gradient (26). These results are somewhat in contrast to ours but highlights the importance of the scale of investigation—that is, the plots from this recent study were within the same climate zone within 40 km, whereas our study spans different climate zones and ca. 1,000 km distance.

Taken together, the environment defines who can grow where (potential niche), and the moss host exerts another selection pressure that leads to the microbiomes we detect (realized niche) that includes competition, facilitation, etc. Both processes are deterministic mechanisms of community assembly and have been found to be more important for plant-associated microorganisms than stochastic processes such as drift in a global assessment (27).

### Responses of moss-associated nitrogen fixation and microbial community composition to nitrogen additions

The addition of ammonium nitrate significantly decreased N fixation rates and thereby aligned diazotroph activity of boreal and temperate mosses, confirming our third hypothesis. The rate of ammonium nitrate added to the boreal samples was chosen to correspond to the rate of N deposition at the temperate site in Denmark (ca. 15 kg ha^−1^ yr^−1^). However, N fixation was even lower in the boreal mosses treated with ammonium nitrate than in the temperate mosses, possibly because the ammonium nitrate was added only three times and during a short period, while the temperate N deposition rate takes place over a year. Furthermore, the results of the temperate mosses reflect N fixation responses to N over long time, while our N addition results reflect short-term effects, which could be reverted over time (6).

In the mosses treated with ammonium nitrate, N fixation was lower in *H. splendens* than in *P. schreberi* and *Sphagnum* sp., suggesting that sensitivity toward increased N loads is not universal and depends on moss hosts and associated diazotrophs (6). Different responses toward N additions were also seen in the microbiome associated with the three mosses. While the microbiome of *Sphagnum* sp. from the boreal forest became more similar after N addition to the microbiome of the temperate *Sphagnum* moss, this pattern was not seen for the other two moss species. The microbiome of *H. splendens* did not change after N additions, and for *P. schreberi*, they completely changed but did not align with the temperate microbiome but became more similar to the microbiome of *Sphagnum* sp. treated with N. Thus, we cannot confirm our third hypothesis that the microbiomes of the boreal mosses would align with the temperate mosses after N additions. This indicates that N status of the environment is not the only driver of this. Indeed, climate and pH are at least as strong drivers of microbial community composition than nutrient availability is, including on mosses (10). The lack of a change in the microbiome of *H. splendens* after N additions is surprising and hints to a strong control of the moss host over the associated microorganisms or a strong buffering capacity of the moss host toward environmental change. However, in contrast, N fixation was inhibited drastically after N additions in the same moss. This highlights that changes in functions (N fixation) do not have to translate into structural changes in the microbiome. Certain taxa disappeared from the mosses after N additions, such as Cyanobacteria from *P. schreberi*, while others proliferated (e.g., *Bacillota* associated with *Sphagnum* sp. and *P. schreberi*). A reduction in diazotroph numbers on mosses upon N addition has been observed earlier and is likely due to the high cost of maintaining N fixation activity when N is available. Indeed, mosses excrete compounds to attract diazotrophs from soil when they are in need of N (28).

### Conclusions

Both the forest type and host species shape the microbiome and N fixation associated with mosses, and N availability can exert a strong influence on associated N fixation and microbiomes. However, a drastic reduction in N fixation rates as a result of N input does not automatically translate into changes in moss-associated microbial communities even though certain diazotrophs disappeared from the mosses. While the environment defines the potential niche of microbiomes, the host species becomes more important in shaping associated microbiomes with increasing phylogenetic distance of the moss host. Additionally, other potential diazotrophs, such as members of *Pseudomonadota* and *Hyphomicrobiales,* were abundant on the mosses, expanding our view on moss-microbe interactions and highlighting that N fixation is not only cyanobacteria-driven but also the outcome of multiple taxa.

## MATERIALS AND METHODS

### Site descriptions

The three moss species (*Hylocomium splendens*, *Pleurozium schreberi*, and *Sphagnum* sp.) were collected in a temperate forest north of Copenhagen, Denmark (Bøllemosen), and a boreal forest in northern Sweden (Varjisån). The temperate forest is located at latitude 55.83°N, longitude 12.56°E, and 15 m above sea level. The mean annual precipitation is approximately 728 mm, and the mean annual temperature is approximately 9.0°C (https://en.climate-data.org/europa/danmark/capital-region-of-denmark/skodsborg-72960/). Bøllemosen is a natural area that includes a peat bog, a small lake, and a surrounding temperate forest, primarily inhabited by *Betula* trees and shrubs such as *Calluna*, *Vaccinium myrtillus*, and *V. oxycoccus*. The boreal forest site (Varjisån) is located at latitude 66.01°N and longitude 19.51°E and between 230 and 540 m above sea level. The mean annual precipitation is approximately 570 mm, and the mean annual temperature is approximately 1°C (29). The forest vegetation contains shrubs such as *Empetrum hermaphroditum*, *Vaccinium vitis-idaea*, and *V. myrtillus*; forbs and herbs such as *Actaea spicata*, *Betula pubescens*, *Geranium sylvaticum*, *Gymnocarpium dryopteris*, *Maianthemum bifolium*, *Paris quadrifolia, Rubus spectabilis*, and *Solidago virgaurea*; and trees such as *Betula pubescens*, *Picea abies*, *Pinus sylvestris*, *Sorbus aucuparia*, and *Rubus spectabilis* (26, 29).

### Moss sampling

Six replicates of three moss species (*Hylocomium splendens*, *Pleurozium schreberi*, and *Sphagnum* sp.) were collected by hand in Bøllemosen in August 2024 from different areas of the forest and at least 5 m apart from each other. The mosses were transferred to transparent plastic bags and transported back to Copenhagen and sorted shortly after. In Varjisån, 12 replicates of *Sphagnum* sp. and 24 replicates each of *H. splendens* and *P. schreberi* were collected in June 2024 following the same procedure. The number of samples reflected their distribution across the forest, where *H. splendens* and *P. schreberi* were available in larger areas. We collected more replicates in the boreal forest than in the temperate forest because we aimed to add N to half of the samples. All moss samples were cleaned from any adherent soil particles and soaked in double-distilled water (ddH_2_O) for 25 min to ensure water saturation before they were sorted into 20 mL glass vials (ca. 1 g fresh weight per sample). Samples were kept in an Aralab FitoClima 1200 PLH Plus growth chamber (ARAlab, Rio de Mouro, Portugal) at 20°C, 150 μmol photons m^−2^ s^−1^ photosynthetically active radiation, 80% humidity, and a 12/12 h light/dark cycle. During the course of the experiment, the mosses were sprayed with ddH_2_O as needed to prevent drying out.

### Nitrogen additions

The N deposition in Bøllemosen is approximately 15 kg N ha^−1^ yr^−1^. To be able to assess if N fixation and microbial communities associated with mosses from the boreal forest align with the ones in the temperate forest, we added an amount of N corresponding to this rate to the mosses from the boreal forest. To do this, a 4.49% ammonium nitrate (NH_4_NO_3_) stock solution was prepared, and 1 mL of the stock solution was added to half of the samples from the boreal forests. At the same time, 1 mL of double-distilled water was added to the other half of the samples from the boreal forest serving as controls. After that, the vials were placed in the growth chamber (settings the same as stated above). The N addition was repeated with the stock solution once a week during the course of 3 weeks, that is, 1/3 rate of the final amount of ammonium nitrate per addition. This was done to get as natural results as possible and to not stress the mosses and their microbiomes too much at once. After the final ammonium nitrate addition, the samples were immediately incubated for acetylene reduction assay measurements.

### Nitrogen fixation measurements

Nitrogen fixation was measured as nitrogenase activity with the acetylene reduction assay (ARA). In the acetylene reduction assays, acetylene (C_2_H_2_) is reduced to ethylene (C_2_H_4_) via the nitrogenase enzyme and is used as a proxy for N fixation activity (30). The mosses in the 20 mL glass vials were sealed with a rubber septum, and 2 mL of air was replaced with acetylene gas using sterile syringes and needles (corresponding to 10% acetylene per glass vial). Along with this, three vials containing 10% acetylene gas only were also incubated and analyzed to account for any residual ethylene presence in the acetylene gas, which was subtracted from the results. Following this, the samples were incubated in the same conditions described above. After 19–20 h of incubation, ethylene production was analyzed on an Agilent 8890 Gas Chromatograph (GC) coupled to an automatic headspace sampler and equipped with a J&W CarboBOND column (Agilent Technologies, Santa Clara, USA), oven temperature at 60°C and pressure set to 10 psi. The measurements were conducted along with a set of vials containing ethylene gas standards. After gas chromatography was completed, the rubber septa were removed, and the vials were returned to the growth chambers for further incubations. Acetylene reduction assays were performed shortly after the N additions (3 weeks after; see below).

### 16S rRNA gene sequencing

After the three weeks of N additions, we froze the samples at −20°C freezer for subsequent DNA extraction and analysis of the moss-associated bacterial communities. All samples were freeze-dried in a Christ Alpha 1-4 LDplus freeze (Christ, Søborg, Denmark) dryer at a temperature of −55°C. After the freeze-drying, the total dry weight was recorded in order to present values on a per dry mass basis. Freeze-dried mosses were crushed into a fine powder in liquid N with sterile metal spatulas. The DNA extractions were performed using the DNeasy PowerSoil Kit (Qiagen, Hilden, Germany) following the manufacturer’s instructions. The isolated DNA was used for the PCR amplification of the V3-V4 region of the 16S rRNA gene using the 341F (5′-CCTAYGGGRBGCASCAG-3′) and 806R (5′-GGACTACNNGGGTATCTAAT-3′) (31) primer pair. The amplicons were used for generating 250 bp paired-end read data sets with the Illumina HiSeq sequencer (Illumina, San Diego, USA). DNA quality control, PCR amplification, and sequencing were performed by Novogene Europe (Munich, Germany).

### Data analysis

Data analysis and visualization of the N fixation data were conducted with R. v. 4.4.1 using RStudio v. 2024.09.0 + 375. Data were tested for normality using the Shapiro-Wilk test and for homogeneity of variance using Levene’s test. If the data were not normally distributed, log transformation was performed. Since the ethylene production rates were still not normally distributed, Mann-Whitney *U* test (32) was run to test for differences in ethylene production between the two forest types, and Kruskal-Wallis tests (33) were performed to test for differences in ethylene production between moss species and between the N additions. They were followed by Dunn’s post-hoc tests (34) with Holm-Bonferroni corrections as a multiple test procedure (35). This procedure controls the family-wise error rate (FWER) and corrects for type I errors without being too conservative, making it suitable for ecological studies, where many effects are moderate/weak (36). Furthermore, the relationship between the relative abundance of orders with diazotrophs and ethylene production was assessed with Spearman correlation. Summarization and filtering of data were performed using the *dplyr* v. 1.1.4 package (37). Visualizations and bar plots were made using the *ggplot2* v. 3.5.1 package (38), and significant results from the tests were visualized using the *ggsignif* v. 0.6.4 package (39).

The DNA sequencing data were analyzed using the QIIME2 pipeline v. 2024.10 (40). Quality control and denoising were achieved using QIIME2’s q2-dada2 plugin with its standard parameters, which performs Phred quality filtering, chimera checking, and paired-end read joining as well as amplicon sequence variant (ASV) selection using the DADA2 algorithm (41). For the taxonomic identification of the resulting ASVs, we used Scikit-learn (42) and the Silva database v. 138 (43) using QIIME2’s q2-feature-classifier plugin. The ASVs were aligned with MAFFT (44), and a maximum likelihood phylogenetic tree was reconstructed with FastTree (45, 46) via the q2-phylogeny plugin. The data were normalized via rarefaction curves with a sampling depth of 1,000 and used to calculate diversity metrics using the q2-diversity plugin, which evaluates differences between treatments in alpha- and beta-diversity analyses with Kruskal-Wallis and PERMANOVA tests, respectively. The Bray-Curtis distance metric was used to calculate beta diversity and generate principal coordinate analyses (PCoA) with Emperor via the q2-emperor plugin. Since we identified only two of the three selected mosses to species level, we ran the statistical tests with and without all three moss species to answer the question on host vs environmental influence only with the two identified mosses.

## Data Availability

The sequences used in this work have been deposited in the Sequence Read Archive (SRA) database of the National Center for Biotechnology Information (NCBI) under accession number PRJNA1353845.

## SUPPLEMENTAL MATERIAL

Figure S1 aem.00828-26-s0001.pdf Taxonomic bar plots of microbial phyla associated with moss samples.

ASM does not own the copyrights to Supplemental Material that may be linked to, or accessed through, an article. The authors have granted ASM a non-exclusive, world-wide license to publish the Supplemental Material files. Please contact the corresponding author directly for reuse.
