# Supplementary material for: Nitrogen deposition homogenizes moss-microbiomes and associated nitrogen fixation but with host-specific responses
Source: Appl Environ Microbiol. 2026 Jun 15;92(7):e00828-26. doi: 10.1128/aem.00828-26 (PMC13390347; doi:10.1128/aem.00828-26)
Supplement: Figure S1 — Taxonomic bar plots of microbial phyla associated with moss samples. [file aem.00828-26-s0001.pdf]

S1. a) Taxonomic bar plot of the microbial phyla found in all the samples, sorted by the forest and addition groups (control=boreal mosses, ammonium nitrate=boreal ammonium nitrate treated mosses and untreated=temperate mosses) and species. b) Taxonomic bar plot of the two common diazotrophic phyla, *Pseudomonadota* and *Cyanobacteriota*, found in the samples, sorted the forest and addition groups (control=boreal mosses, ammonium nitrate=boreal ammonium nitrate treated mosses and untreated=temperate mosses) and species.

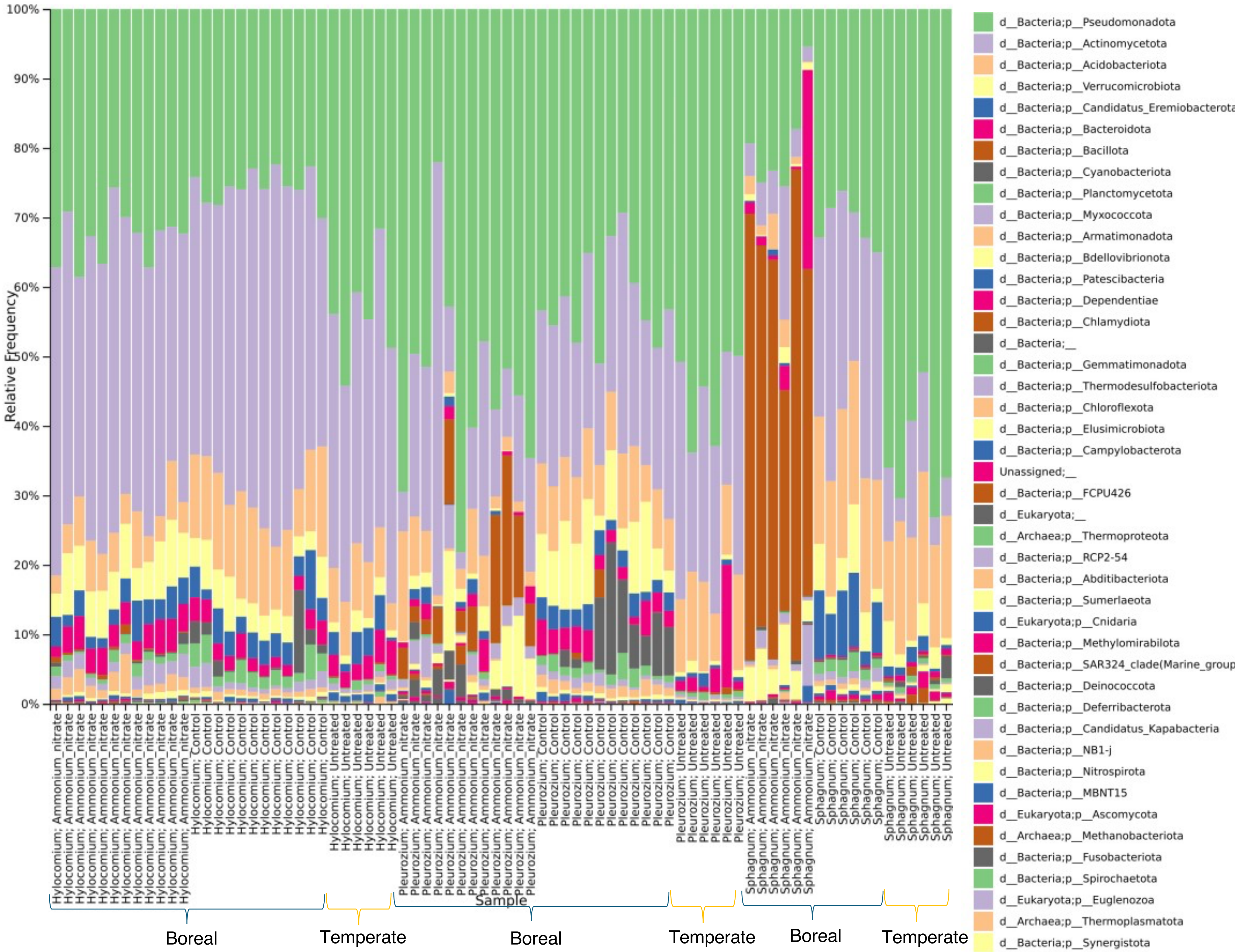
100%
90%
80%
70%
60%
50%
40%
30%
20%
10%
0%
Relative Frequency
Sample
Hylocomium; Ammonium_nitrate
Hylocomium; Control
Hylocomium; Untreated
Pleurozium; Ammonium_nitrate
Pleurozium; Control
Pleurozium; Untreated
Sphagnum; Ammonium_nitrate
Sphagnum; Control
Sphagnum; Untreated
Boreal
Temperate
Boreal
Temperate
Boreal
Temperate
d__Bacteria;p__Pseudomonadota
d__Bacteria;p__Actinomycetota
d__Bacteria;p__Acidobacteriota
d__Bacteria;p__Verrucomicrobiota
d__Bacteria;p__Candidatus_Eremiobacterota
d__Bacteria;p__Bacteroidota
d__Bacteria;p__Bacillota
d__Bacteria;p__Cyanobacteriota
d__Bacteria;p__Planctomycetota
d__Bacteria;p__Myxococcota
d__Bacteria;p__Armatimonadota
d__Bacteria;p__Bdellovibrionota
d__Bacteria;p__Patescibacteria
d__Bacteria;p__Dependentiae
d__Bacteria;p__Chlamydiota
d__Bacteria;__
d__Bacteria;p__Gemmatimonadota
d__Bacteria;p__Thermodesulfobacteriota
d__Bacteria;p__Chloroflexota
d__Bacteria;p__Elusimicrobiota
d__Bacteria;p__Campylobacterota
Unassigned;__
d__Bacteria;p__FCPU426
d__Eukaryota;__
d__Archaea;p__Thermoproteota
d__Bacteria;p__RCP2-54
d__Bacteria;p__Abditibacteriota
d__Bacteria;p__Sumerlaeota
d__Eukaryota;p__Cnidaria
d__Bacteria;p__Methylomirabilota
d__Bacteria;p__SAR324_clade(Marine_group
d__Bacteria;p__Deinococcota
d__Bacteria;p__Deferribacterota
d__Bacteria;p__Candidatus_Kapabacteria
d__Bacteria;p__NB1-j
d__Bacteria;p__Nitrospirota
d__Bacteria;p__MBNT15
d__Eukaryota;p__Ascomycota
d__Archaea;p__Methanobacteriota
d__Bacteria;p__Fusobacteriota
d__Bacteria;p__Spirochaetota
d__Eukaryota;p__Euglenozoa
d__Archaea;p__Thermoplasmatota
d__Bacteria;p__Synergistota

a)

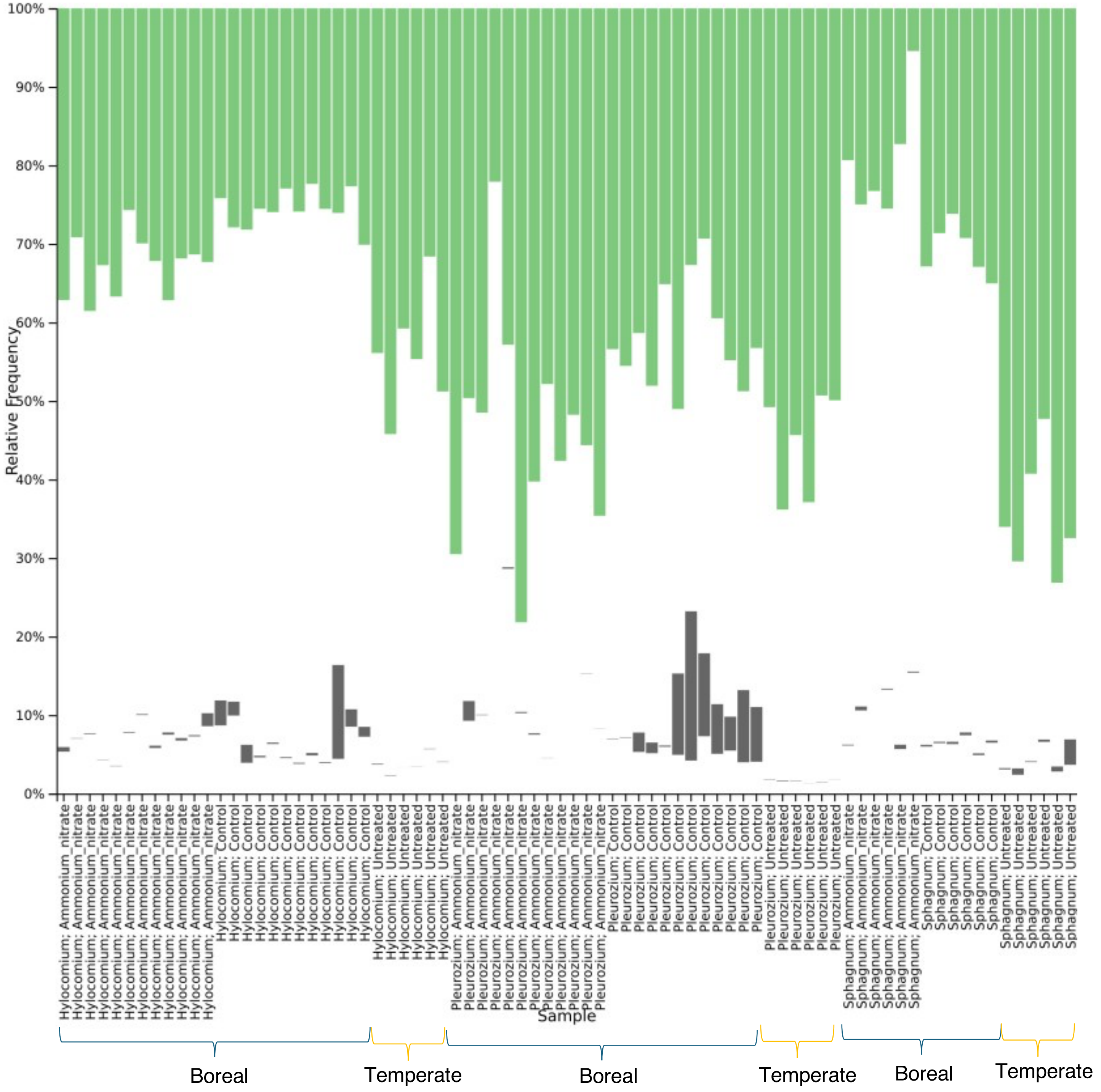
100%
90%
80%
70%
60%
50%
40%
30%
20%
10%
0%
Relative Frequency
Hylocomium; Ammonium_nitrate
Hylocomium; Control
Hylocomium; Untreated
Pleurozium; Ammonium_nitrate
Pleurozium; Control
Pleurozium; Untreated
Sphagnum; Ammonium_nitrate
Sphagnum; Control
Sphagnum; Untreated
Sample
Boreal
Temperate
Boreal
Temperate
Boreal
Temperate

b)
